# Comparative transcriptomic analysis identifies genes responsible for fruit count and oil yield in the oil tea plant *Camellia chekiangoleosa*

**DOI:** 10.1038/s41598-018-24073-z

**Published:** 2018-04-27

**Authors:** Yun Xie, Xuewen Wang

**Affiliations:** 1 Jiyang College, Zhejiang A&F University, Zhuji, Zhejiang, 311800 China; 2 Department of Genetics, University of Georgia, Athens, 30602 USA

## Abstract

Fruit yield is an important trait for horticultural crops. However, the limited fruit yield of *Camellia chekiangoleosa*, a novel promising oil tree, restricts the production of oil. The breeding improvement is little due to its long generation time and lack of available genomic sequence. We identified distinct fruit count phenotypes, high-yield (HY) and low-yield (LY). To understand the underlying genetic basis, we focused on global gene discovery and expression levels in floral buds, which affect fruit count. A total of 140,299 *de novo* assembled unigenes were obtained using RNA-seq technology, and more genes were expressed in HY than in LY. 2395 differentially expressed genes (DEGs) were identified and enriched in membrane, energy metabolism, secondary metabolism, fatty acid biosynthesis and metabolism, and 18 other metabolic pathways. Of the DEGs, twelve identified transcription factors, including AP2, mostly involve in inflorescence and flower development and in hormone networks. Key DEGs in fatty acid biosynthesis (Fab) *FabB*, *FabF*, *FabZ*, and *AccD* were highly expressed in floral buds and associated with high oil yield in fruits. We hypothesized that a potential link exists between fruit count and its oil yield. These findings help to elucidate the molecular cues affecting fruit count and oil yield.

## Introduction

*Camellia* is a genus of evergreen flowering plants in the family Theaceae. *C. sinensis* and *C. oleifera* are the top two most important species in this genus, because the *C. sinensis* leaf is used for making drinking tea while the *C. oleifera* seed is used for producing tea seed oil. *C. chekiangoleosa* is a close relative of *C. oleifera* that produces a higher quality of tea seed oil. Both *C. chekiangoleosa* and *C. oleifera* are called oil tea plants, although their phylogenetic relation is still uncertain. Tea seed oil is known as one of the top four major woody oils after palm, olive, and coconut. As one of the healthiest vegetable oils, with up to ~80% unsaturated fatty acids^1^, tea seed oil has been emerging as one of the most expensive oils for cooking and medicinal use. However, a bottleneck in industrial tea seed oil production is the very limited availability of seeded fruits. Researchers and breeders are interested in improving the oil production of oil tea fruits^2^. Recent advances in other crops, such as corn, demonstrate that an understanding of genomic and genetic bases can speed up molecular breeding for improvement in preferred traits such as yield^3^.

The current knowledge of the genomics and genetics of oil tea plants is very little. To date, the whole genomic sequence is still unknown, although the draft genome of a closely related species, *C. sinensis*, became publicly available recently^4^. However, only some short reads of transcripts have been reported for *C. oleifera* and none for *C. chekiangoleosa*. For example, some transcripts in *C. oleifera* were generated from the 454 GS-FLX sequencing platform^5^. Most of the available *C. oleifera* reads are Illumina short reads, including leaf transcriptomic data^6,7^. A National Engineering and Technology Research Center of Camellia Oil has been funded by the Chinese government to start an evaluation of oil tea plant germplasm, with the aim of helping to develop new cultivars. The minimum duration from a seedling to a fruit of an oil tea plant is approximately 5–7 years, and the full generation time of an oil tea plant is 10–120 years^8^, which suggests an extremely long cycle for genetic improvement. Intriguingly, molecular investigation of these oil trees could reduce the breeding cycle by providing molecular cues as selection markers. However, the genetic background and origin of oil tea plants is unknown, although it has been recorded that several varieties of oil tea plants have very different phenotypes^8^.

The yields of fruits or seeds in *Camellia* vary greatly depending on the germplasm^2^. A high yield of fruit count per tree is a preferred trait and may be the best solution for the tea seed oil industry. The number of fruits is determined by the number of flowers, rate of successful pollination, fruit set and fruit abortion before harvesting. The plant inflorescences develop from floral meristems and are modulated by its stereotypical arrangement and environmental cues^9^. Both phytohormones and genes expression in flowers and fruits affect fruit yield^10^. The transcription factors such as LEAFY, FLOWERING LOCUS genes, and transcription factors in the MADS box family, such as SOC1, FRUITFULL and APETALA1^9^ have been known to also affect fruit yield. An accumulation of auxin in the floral primordia and cytokinin could induce the development of flowers through regulation of the expression of a series of genes^10^. The sugar metabolism and phytohormones, e.g., auxin, gibberellin, and ABA, in floral buds affect ovule-wall cell division and male fertility, which determines fruit set^11^. It is known that auxin and gibberellin play roles in fruit set in crops such as tomato^12^ and citrus^13^. However, little is known about fruit set in oil tea plants. Cellular structural investigation revealed that self-incompatibility just after fertilization and before zygote division may affect seed set in some *C. oleifera*^14^. Some oil tea varieties are self-compatible or partially self-compatible. The expression levels of the genes *rbcL* and *rbcS* were reported to strongly and positively correlate with seed-oil yield in *C. oleifera*, and thus, these genes may be candidate markers for the selection of high seed-oil yield^2^. Some expressed genes in *C. oleifera* are available^5^, but these genes were obtained from very low read depth. With advances in RNA-seq, an increasing number of reports have demonstrated that gene expression can associate with traits or phenotypes in many plants^15–17^. In *C. oleifera*, putative homologous unigenes in leaves were identified for some traits by using RNA-seq, e.g., unigenes for cold tolerance across latitudes^6^ and unigenes for drought^7^. To the best of our knowledge, no report on either *C. oleifera* or *C. chekiangoleosa* has investigated the genetic control of fruit yield at the global gene expression level. Therefore, a whole-transcriptome investigation of gene expression in the floral buds will provide clues to understand the effects of gene expression on fruit count. The importance of a similar study has been highlighted in oil palm^18^.

In this study, we identified two distinct types of trees from a population at the likely centre of diversity of *C. chekiangoleosa*, one with high yield (HY) and one with low yield (LY) with respect to fruit count for many years. This *C. chekiangoleosa* variety has larger red flowers and larger fruits than those previously reported for *C. oleifera*^2,5,14^. Elucidating the molecular mechanisms in floral buds, which are responsible for fruit count in *C. chekiangoleosa*, is fundamental to our understanding of reproductive biology in this species and our ability to design improvements in crop yield. To achieve this aim, we focused on gene discovery and the differences in gene expression in the floral buds of *C. chekiangoleosa*. We compared the gene expression profiles at the whole-transcriptome level using RNA-seq data from three trees with HY or LY phenotypes and identified candidate genes which may contribute to the observed fruit count and the measured oil yield per fruit. This study revealed the molecular basis of HY and LY *C. chekiangoleosa*, and the results may help future breeding improvement.

## Results

### Two distinct phenotypes of fruit yield

**Figure 1:**
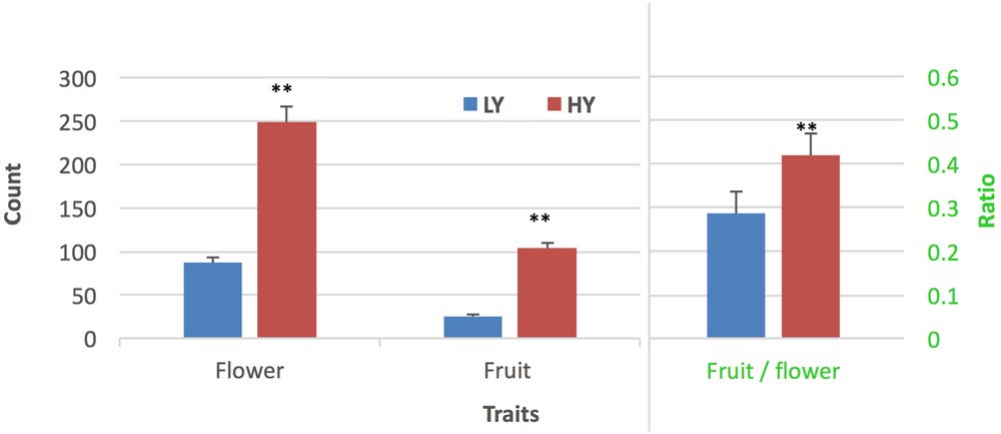
*Camellia chekiangoleosa* trees with two distinct fruit yields. Image shows the comparison of the flower count, fruit count and ratio of fruit/flower count. **Represents significance at *p* < 0.01 by two-way statistical ANOVA analysis.

We identified two distinct types of fruit count, HY and LY, in a *C. chekiangoleosa* tree population planted 20 years ago in the same location with GPS latitude 29.978633–29.978821 and longitude 118.966596–118.967638. The fruit yield phenotypes were consistent through many years. To understand whether the yield differences resulted from the number of flowers or fruits, we recorded the flower count and fruit count of each tree in three biological replicates for three consecutive years. A comparison showed that the HY type had significantly more flowers and fruits than the LY type did (*p* < 0.01) (Fig. 1, Supplementary Table S1), indicating that both higher flower number and higher fruit number account for the high fruit yield. To check the successful rate of fruit set from flowers, we compared the ratios of fruit number to flower number and found that the ratio was significantly higher in the HY type than the LY type (*p* < 0.01) (Fig. 1, Supplementary Table S1). A two-way ANOVA statistical analysis showed that the HY and LY traits did not associate with the year factor. Flowering time may affect the fruit yield^3^, but we did not find significant differences in flowering time between the two types of trees; both types bloomed between February and March. We also did not find significant differences (*p* < 0.05) in leaf size, leaf thickness or leaf chlorophyll content between the HY and LY types (Table 1). These indicated that no obvious morphological difference could explain the difference in fruit yields. Together, these results suggested that the observed fruit yield traits in *C. chekiangoleosa* were controlled genetically. It is known that gene expression and phytohormones in floral buds could affect fruit yield^9–11^; therefore, we focused on the differences of gene expression in the floral buds.

**Table 1:** Comparison of leaf phenotypes and chlorophyll contents between LY and HY trees.

Plant ID	Chlorophyll (absorbance)		Leaf area (cm^2^)		Leaf thickness (mm)	
	LY	HY	LY	HY	LY	HY
1	68.8	70.1	40.2	47.7	0.54	0.60
2	70.3	69.3	41.3	48.0	0.59	0.60
3	71.3	72.2	36.2	38.4	0.59	0.58
Mean	70.2	70.5	39.2	44.7	0.60	0.60
S.E.	0.7	0.8	1.6	3.2	0.02	0.01
Significance	Not		Not		Not	

Data for each individual plant was measured from at least 10 leaf samples. The chlorophyll content was measured using the chlorophyll meter SPAD-502Plus (Konica Minolta Optics, Inc.). S.E. represents the standard error. The T-test statistical analysis was used to test the significance (*) of the differences at p < 0.05.

### *De novo* assembled transcripts and transcriptome profiles of *C. chekiangoleosa*

To understand the molecular differences between the floral buds at the global level of gene expression, we first examined the transcriptome using RNA-seq technology. At least ten fresh floral buds of each of three LY or HY trees were collected in Dec 2015, when they had already reached the maximum size of floral buds and would blossom after 2–3 months. The transcripts present in the floral buds of each tree were deeply sequenced independently on the Illumina HiSeq. 4000 platform. A total of 361 million paired-end 125- or 150-bp reads were generated with a quality score (Phred Q30) >90% (Supplementary Table S2). Due to the lack of a reference sequence for the *C. chekiangoleosa* genome, all reads were pre-processed for quality control, merged, and *de novo* assembled into transcripts using Trinity software^19,20^ (version 201308), and 140,299 unigenes with a GC content of 40% were obtained. More than 75% of the paired-end reads were properly mapped back to the unigenes, indicating a good transcript assembly. The RNA-seq reads had 485X coverage of the unigene set. The average length of unigenes was 586 bp (Supplementary Table S2). The RNA-seq reads from each sample, including the lowest 17 million reads, were enough to sensitively detect more than 80% of the assembled unigene set with more than 0.1 fragments per kilobase length per million reads (FPKM) expression level. The sequences of the RNA-seq reads and assembled unigenes were deposited and are publicly available at the Short Read Archive and DDBJ/ENA/GenBank under the BioProject accession numbers PRJNA415233 and GFZM00000000, respectively. The version described in this report is version GFZM02000000.

### Functional annotation of unigenes

The assembled unigenes were annotated by similarity searching (E-value ≤ 10 for Pfam using HMMER3, E-value ≤ 10^−5^ and identity >90% using BlastX) against the databases NCBI NR, KOG, Pfam, and UniProtKB using the methods described previously^16^. A total of 71,167 (50.7%) unigenes were annotated at least once from these databases (Supplementary Table S2). The highest number of unigenes, 61,059 (43.5%), were annotated against the NR database. A total of 41,936 (29.9%) unigenes were functionally annotated in the eukaryotic orthologous database KOG. In addition, 32,356 (23.1%) and 50,914 (42.9%) unigenes were annotated against the manually curated Swiss-Prot database and the automatically annotated TrEMBL database at UniProtKB, respectively. Based on all the annotation information, we obtained 50,914 Gene Ontology (GO) terms (Supplementary Table S2).

### Gene expression profiles and differentially expressed genes in floral buds

**Figure 2:**
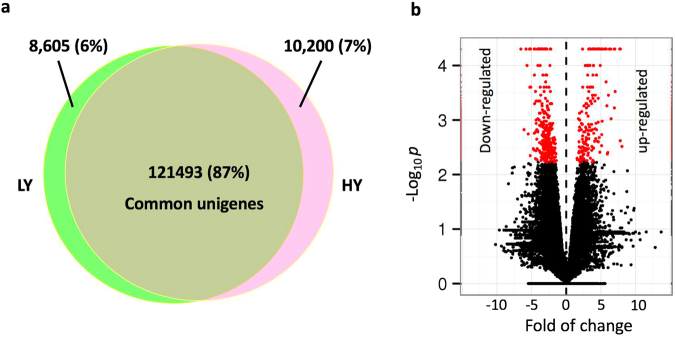
Distribution of expressed unigenes in two types of *Camellia chekiangoleosa*. Image a shows the commonly or specifically expressed unigenes. Image b shows the differentially expressed genes (red) with expression changes of more than 2-fold, *p* < 0.05 and *q* < 0.001 (hypergeometric test). The fold change was relative to the expression level in the LY type after the log_2_(FPKM) calculation. HY and LY represent high and low yields based on fruit count.

To examine the number of expressed genes and their levels, we calculated the transcript abundance in each sample in FPKM. We identified 131,693 and 130,098 expressed genes (FPKM > 0) with lengths greater than 200 bp in the HY and the LY types, suggesting that more genes (1595) were expressed in HY trees (Fig. 2a). The median expression level was also higher in the HY type (4.65 FPKM) than in the LY type (4.13 FPKM).

We compared the gene expression levels in both the LY and HY types, and revealed 2,395 differentially expressed genes (DEGs) with at least 2-fold changes in abundance at statistical cutoffs of *p* < 0.05 and *q* < 0.001 (hypergeometric test) using the LY type as the control (Fig. 2b, Supplementary data 1). Among these DEGs, 819 and 1576 were up- and down-regulated in the floral buds (Fig. 2b, Supplementary data 1), suggesting that more genes were down-regulated in the HY type and accounting for the difference in fruit yield.

**Figure 3:**
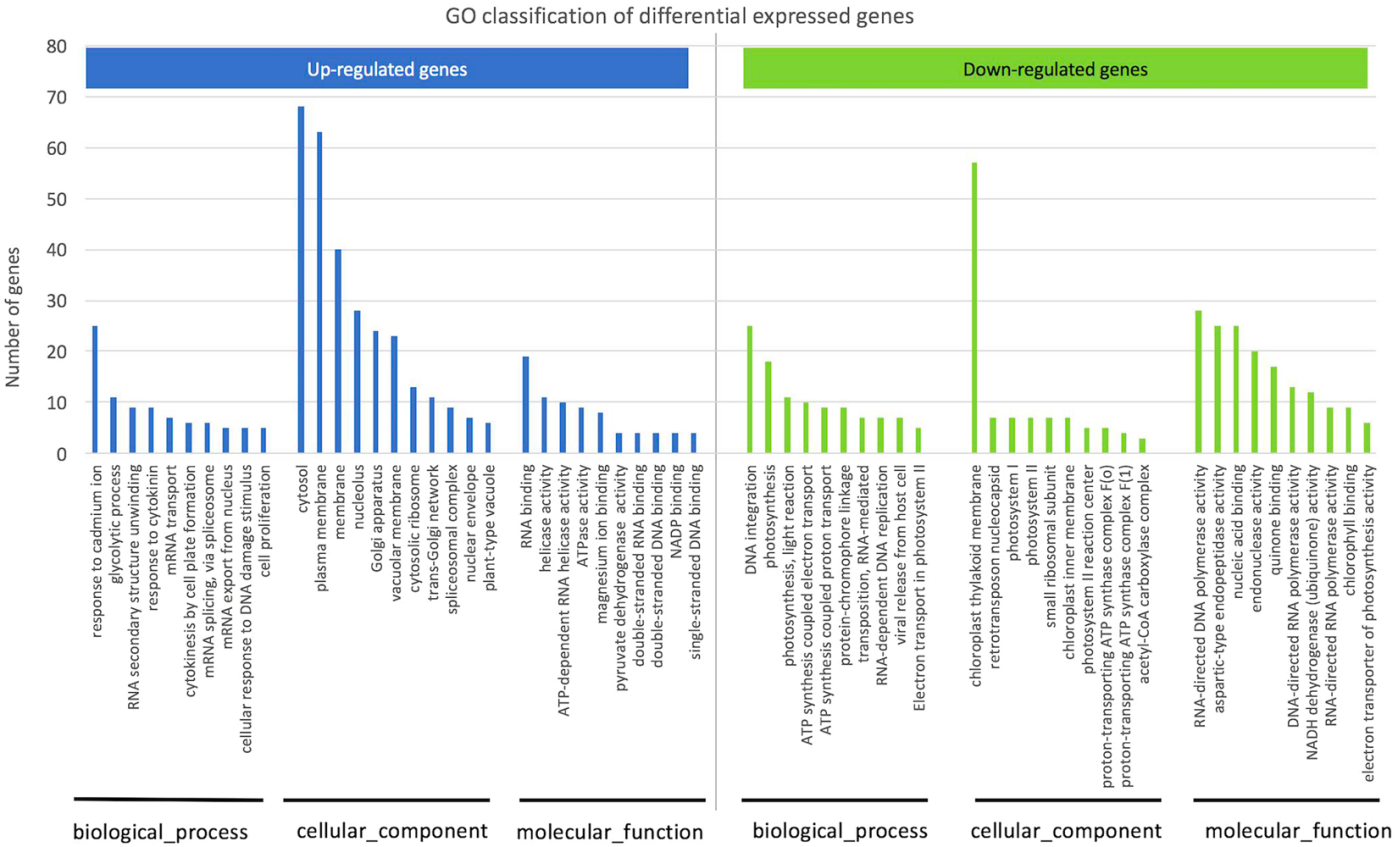
Top enriched GO terms of differentially expressed genes. Image shows the top enriched GO terms of the DEGs ranked by the number of genes. Only the top ten up- or down-regulated DEGs in each category (biological process, cellular component and molecular function) are shown.

To classify the functions of the identified DEGs, we conducted a GO analysis against GO term databases (http://www.geneontology.org). The results showed that the up- and down- regulated DEGs were enriched in 267 and 138 GO classifications (hypergeometric, *p* < 0.05), respectively (Supplementary Table S3). Among the up-regulated DEGs, the top GO classifications in the biological process category were enriched in “response to cadmium ion”, “glycolytic process”, “RNA secondary structure unwinding”, and “response to cytokinin”, etc., suggesting that the DEGs were mainly involved in changes in gene activity, cell division and energy metabolism. In the category of cellular component, the top GOs were enriched in “cytosol”, “plasm membrane”, “membrane” and “nucleolus”. In the category of molecular function, the top GOs were enriched in “RNA binding”, “helicase activity”, “ATP-dependent RNA helicase activity” and “ATPase activity” (Fig. 3). In contrast, among the down-regulated DEGs, the enriched GOs in the category of biological process were associated with photosynthesis and ATP synthesis. The enriched GOs in the category of cellular component were enriched in “chloroplast thylakoid membrane”, “retrotransposon nucleocapsid”, “photosystems” and “ATP synthase complex”. The GOs in the category of molecular function were enriched in “RNA-directed DNA polymerase activity”, “aspartic-type endopeptidase activity”, “nucleic acid binding” and “endonuclease activity”. In summary, the GOs suggest that DEGs involved in energy metabolism, membrane, and nucleotide metabolism confer the main differences in the floral buds between the two types.

### Key candidate genes and regulated pathways

**Figure 4:**
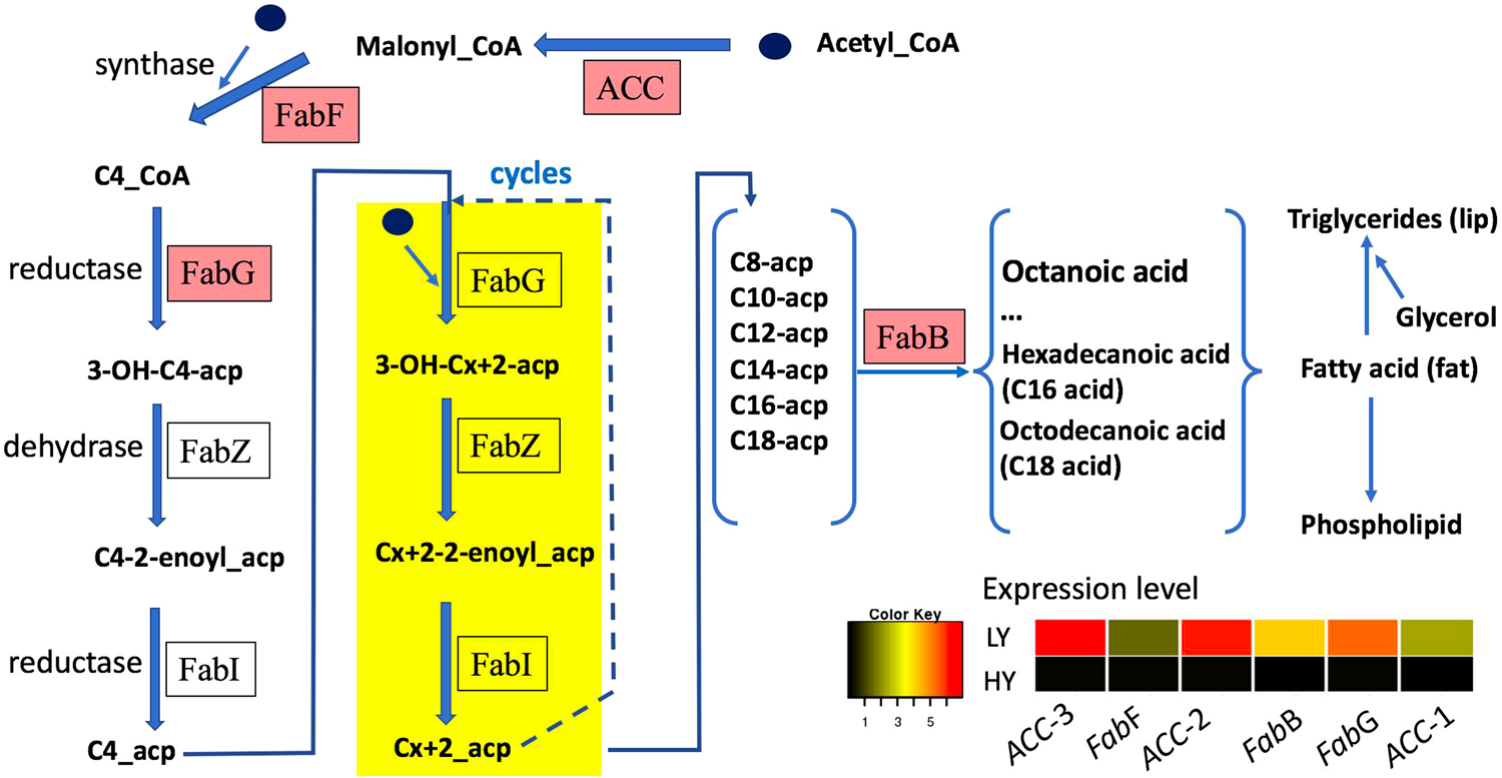
Key genes differentially regulated in the fatty acid synthesis pathway in *C. chekiangoleosa*. Image shows the key enzymes encoded by DEGs highlighted in the pink box in the fatty acid synthesis pathway. The heatmap at the bottom right shows the expression levels of the corresponding DEGs in the pathway. LY and HY represent the low-yield and high-yield phenotypes of *C. chekiangoleosa*. ACC, FabF, FabG and FabB represent the key enzymes acetyl-CoA carboxylase 1 [EC:6.4.1.2], 3-oxoacyl-[acyl-carrier-protein] synthase II [EC:2.3.1.179] (FabF), 3-oxoacyl-[acyl-carrier protein] reductase [EC:1.1.1.100] (FabG), and fatty acyl-ACP thioesterase B [EC:3.1.2.14] (FabB), respectively. *ACC*-1, -2, and -3 represent three different encoding genes.

**Figure 5:**
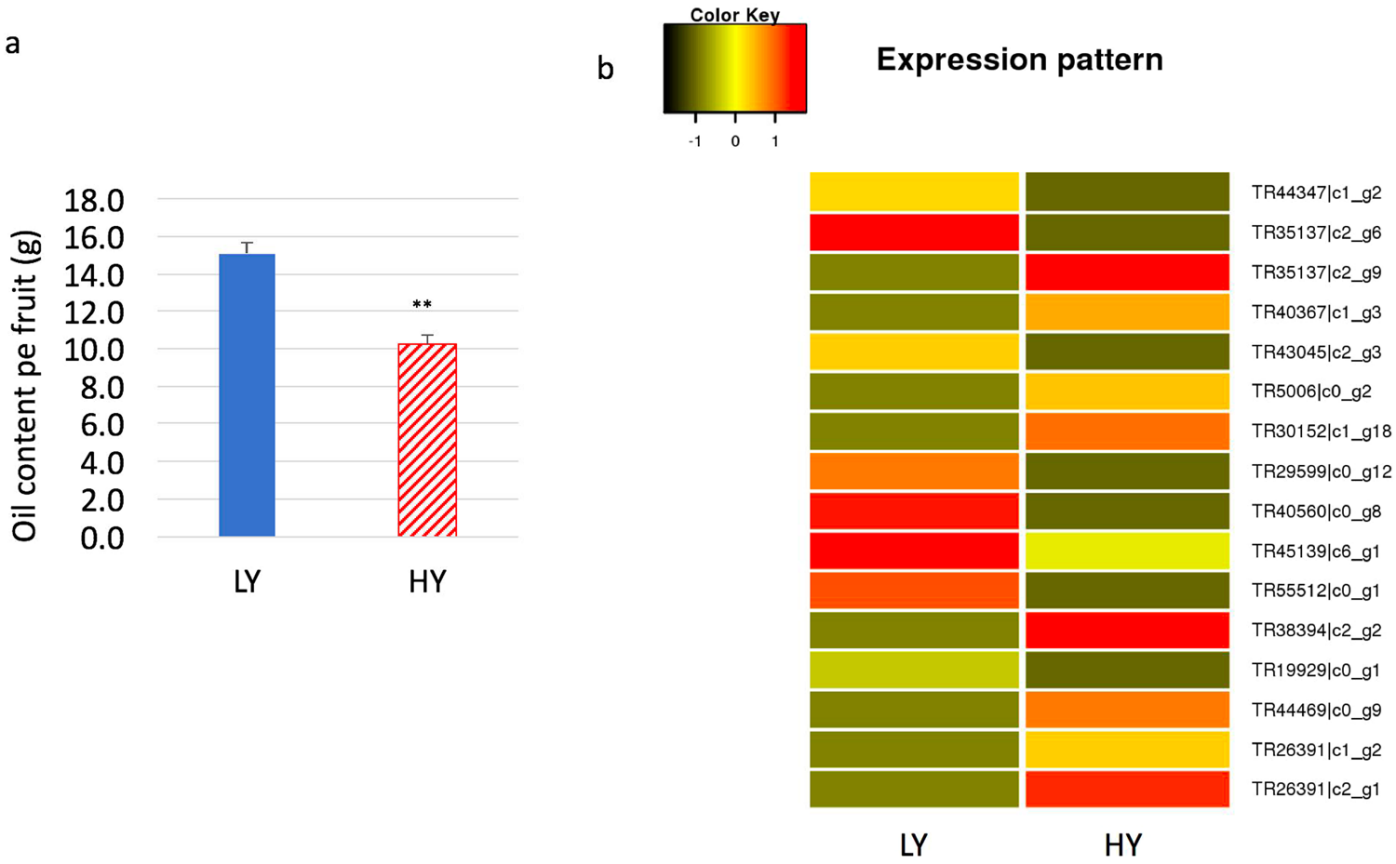
Oil yield and expression levels of transcription factors and regulators among the DEGs. Image a shows the oil yield per fruit. Bars represent the mean value plus error, which was calculated from 10–20 fruits per tree per year with weight normalization relative to HY. Image b shows the expression levels of transcription factors and regulators. LY and HY represent low yield and high yield of fruits in *C. chekiangoleosa*. The name to the right of each line represents the ID of the differentially expressed unigene.

To further investigate whether the DEGs were involved in specific pathways, we searched for homologs of the DEGs among *Arabidopsis* proteins using KAAS^21^ (version 2.1) in the Kyoto Encyclopedia of Genes and Genomes (KEGG at http://www.genome.jp/kegg) to annotate their functions and then mapped the DEGs to available pathways using the KOBAS (version 3) mapper^22^. The results showed that the DEGs were enriched in 18 pathways (hypergeometric, *p* < 0.05 and *q* < 0.05) (Table 2). Among these pathways, the top four were metabolic pathways (140 DEGs), synthesis of secondary metabolites (50 DEGs), oxidative phosphorylation for energy (33 DEGs), and ribosome (30 DEGs). Interestingly, five of the 18 pathways associated with fatty acids were enriched (Table 2). In the fatty acid biosynthesis pathway shared by plants (Fig. 4), the key genes encoding acetyl-CoA carboxylase 1 [EC:6.4.1.2] (ACC) for the initiation and regulation of fatty acid synthesis, 3-oxoacyl-[acyl-carrier-protein] synthase II [EC:2.3.1.179] (FAS2 or FabF), 3-oxoacyl-[acyl-carrier protein] reductase [EC:1.1.1.100] (FabG), and fatty acyl-ACP thioesterase B [EC:3.1.2.14] (FabB) for the final step of synthesis were significantly differentially expressed between the two types of trees. In addition, three DEG copies, TR38934|c2_g3, TR55147|c0_g1 and TR51660|c0_g1, encoding the enzyme ACC were identified. FabF, FabG and FabB are encoded by the unigenes TR41617|c1_g2, TR44078|c2_g7 and TR31380|c0_g3, respectively. The higher expression levels of these fatty-acid-synthesizing DEGs implied that the LY type may have higher oil content than the HY type (Fig. 4). We further tested the oil content per fruit across three years and revealed a higher oil content in the LY type than in the HY type (Fig. 5a). These results demonstrated that the higher expression of these fatty-acid-synthesizing DEGs in floral buds positively regulated higher oil content in fruits.

**Table 2:** Metabolic pathways involving the differently expressed genes.

	KEGG pathway name	Homology ID	Enriched DEGs	P value	Q value
1	Metabolic pathways	ath01100	140	1.25E-25	1.13E-23
2	Biosynthesis of secondary metabolites	ath01110	50	0.0001	0.002
3	Oxidative phosphorylation	ath00190	33	1.92E-17	5.77E-16
4	Ribosome	ath03010	30	2.64E-7	3.95E-6
5	Photosynthesis	ath00195	29	1.41E-21	6.33E-20
6	Carbon metabolism	ath01200	22	8.06E-6	9.07E-5
7	Pyrimidine metabolism	ath00240	17	9.10E-8	1.64E-6
8	Purine metabolism	ath00230	15	6.12E-5	0.001
9	Glycolysis/Gluconeogenesis	ath00010	14	1.04E-5	0.0001
10	RNA polymerase	ath03020	13	2.99E-9	6.72E-8
11	Citrate cycle (TCA cycle)	ath00020	8	0.001	0.004
12	Pyruvate metabolism	ath00620	8	0.003	0.02
13	Carbon fixation in photosynthetic organisms	ath00710	7	0.004	0.02
14	Fatty acid metabolism	ath01212	12	1.10E-6	1.41E-5
15	Fatty acid biosynthesis	ath00061	6	0.001	0.01
16	Biosynthesis of unsaturated fatty acids	ath01040	5	0.002	0.01
17	alpha-Linolenic acid metabolism	ath00592	5	0.004	0.02
18	Fatty acid degradation	ath00071	5	0.007	0.04

### Differential expression of transcription factors and regulators

Transcription factors and regulators play important roles in the regulation of gene expression directly and indirectly, respectively. To predict the transcription factors and regulators in the identified DEG list, we conducted data mining using the DEGs against the iTAK database^23^ (version 17.09, http://itak.feilab.net/cgi-bin/itak/index.cgi), which gathers the information of transcription factors in 169 plant genomes. Twelve DEGs were found to belong to known transcription factor families and can be classified into 10 groups, such as PA2/ERF, C2H2, C3H, FAR1, MYB and NAC (Table 3). Four DEGs belonged to the transcriptional regulator category, including GNAT and mTERF. The expression of these transcription factors and regulators showed distinct levels (Fig. 5), which suggested that regulation by transcription factors and regulators could contribute to the differential expression of DEGs in the HY and LY types.

**Table 3:** DEGs annotated as transcription factors and regulators.

Gene ID	Transcription Factors	Group
TR44347|c1_g2	AP2/ERF-ERF	Transcription Factors
TR35137|c2_g6	C2H2	Transcription Factors
TR35137|c2_g9	C2H2	Transcription Factors
TR40367|c1_g3	C3H	Transcription Factors
TR43045|c2_g3	FAR1	Transcription Factors
TR5006|c0_g2	GARP-G2-like	Transcription Factors
TR30152|c1_g18	MADS-M-type	Transcription Factors
TR29599|c0_g12	MYB-related	Transcription Factors
TR40560|c0_g8	MYB-related	Transcription Factors
TR45139|c6_g1	NAC	Transcription Factors
TR55512|c0_g1	NF-YB	Transcription Factors
TR38394|c2_g2	RWP-RK	Transcription Factors
TR19929|c0_g1	GNAT	Transcriptional Regulators
TR44469|c0_g9	mTERF	Transcriptional Regulators
TR26391|c1_g2	Others	Transcriptional Regulators
TR26391|c2_g1	Others	Transcriptional Regulators

## Discussion

The fruit yield of crops, as the most important agricultural trait, has drawn attention from researchers and breeders for a long time. Fruit yield impacts food security and quality. The fruits from *Camellia* oil tea plants are used for producing tea seed oil, which has been accepted as one of the healthiest vegetable oils, similar to the top-ranked olive oil, based on its content of unsaturated fatty acids^1,2^. Low fruit yield has restricted the production of tea seed oil for a long time. Studies and efforts have been made to understand the causes of low fruit yield to improve this trait^2,6,14^. It is known that floral buds affect fruit count in plants^11^. However, none previous study has focused on the effects of floral buds on the fruit yield of *C. chekiangoleosa*, although floral buds could affect the fruit yield in other species^9–11^. In this study, we initially identified the genes expressed in floral buds and then identified the gene expression differences between trees with different fruit yields. In addition, the two phenotypes of fruit count, high-yield and low-yield, were evidenced to be genetically controlled in our study in *C. chekiangoleosa*. Our findings facilitate the understanding of genetic and gene regulatory bases of fruit yield in oil tea plants.

In this study, we found that the fruit count phenotype was directly associated with floral buds and rate of fruit set. HY trees had more flower buds, almost twice as many in HY as in LY, which resulted in more fruits. However, the fruit set ratio was less than 50%, which suggests a commonly low fruit set rate in oil tea plants. A similar scenario is also found in the self-sterile *C. oleifera*, a close relative of *C. chekiangoleosa*^14^. In *C. chekiangoleosa*, the floral buds reach the maximum size in December, while the flowers are in bloom in February and March. This effect could be caused by dormancy that needs to be broken by a cold stimulus. A late dormancy break will reduce fruit yield by affecting hormone levels^24^. Our comparison revealed a higher expressed gene number in the HY type than in the LY type. The 1595 additional expressed genes in the HY type indicated that the high fruit yield trait may come from combined control by multiple genes, most likely not from a single gene. For further research, this trait could be narrowed down to a few genes by combining other methods such as QTL mapping^25^ in the progeny of a cross. However, this study could take at least 20–25 years due to the long-life cycle of *C. chekiangoleosa*. Since these gene expression differences were mined from multiple tree individuals of the LY and HY types, the findings in this study could represent common genes underlying fruit count difference. Of course, the real number of genes may be different from 1595, because *de novo* assembly always results in a high gene number, which is caused by the potential for multiple fragments from a single gene.

Differential gene expression in the HY and LY types could make large contributions to the fruit count trait. We could not determine which of the up- and down-regulated DEGs was the most important contributor to the phenotype in this study, but we did find that important genes were involved in energy metabolism, membrane, and nucleotide metabolism. In addition, we identified five pathways enriched among the DEGs that were associated with fatty acids in the floral buds. This result indicates a strong link between the fatty acid and fruit count phenotypes, which might be due to effects of the hormones produced from the anabolism of fatty acids, such as jasmonate^26^, which affects stamen fertility^27^. The DEGs involved in fatty acid biosynthesis, such as ACC, FabB, FabF, and FabZ, were more highly expressed in the LY type than in the HY type. This indicates high fatty acid biosynthesis in LY, meaning a high level of fatty acid generation in the LY type, which was confirmed by our oil content data. This result is consistent with the common knowledge that oil tea trees with fewer fruits, such as LY, will have a high oil yield from each fruit. As the fruit count is lower in LY, it is possible to deposit more of the carboxyl hydrides fixed by photosynthesis through the reported key genes rbcL and rbcS in the oil tea plant^2^. This finding suggests that the fruit count per tree is low, but the oil yield per fruit is high. Therefore, we should pay attention to both oil yield and fruit yield in breeding practices because of the negative relationship observed between oil yield per fruit and fruit count. However, fruit count has a larger contribution than oil yield per fruit does, meaning that fruit count is the first preference in breeding practice. Our findings also suggest that oil biosynthesis in fruits may be positively predictable from the expression of these fatty acid genes in the floral buds.

Transcription factors are known to regulate floral development^28^ and have proven to be potential gene candidates for improving yield-related traits in crops such as wheat and barley^29^. A previous investigation reported some transcription factors in another oil tea species, *C. oleifera*^5^. In this study, we identified some transcription factors as differently expressed, and further functional verification of the identified factors may help to improve the fruit yield of oil tea in the future, although genetic transformation in oil tea has not been reported. Many of our identified transcription factors among the DEGs, such as the AP2, NAC, NAM, MYB, bHLH, MADS and C2H2 families, have been reported to positively control inflorescence and flower development^28,30^. FLOWERING LOCUS C (FLC), a well-known MADS box gene, delays floral induction by repressing the FLOWERING LOCUS T and SOC1 genes^31,32^. We identified the differentially expressed transcription factor AP2/ERF in the final step of the ethylene signalling pathway. AP2/ERF could function as a regulator to modulate other phytohormone signalling pathways^33^, including that of jasmonate, which is produced from fatty acids^27^. We did not observe DEG-enriched phytohormone pathways, but we found an enriched GO term in “response to cytokinin”, indicating that many phytohormones also play important roles in the difference in the floral buds of LY and HY. Hence, the transcription factors from our study may be of interest for future investigation, for contributing to the HY and LY phenotypes. Photosynthesis and sugar metabolism affect fruit count and yield^2,11^. The top GO functions of our DEGs were enriched in “glycolytic process”, “ATPase activity”, “ATP synthase complex”, and “chloroplast thylakoid membrane”. These functions are closely associated with photosynthesis, sugar and energy, meaning that the GO functions of our DEGs are consistent with those of previous reports^2,11^.

During ecodormancy, epigenetic modification and hormone levels in floral buds could affect flowering and fruit yield^34^. Since the floral bud size had reached its maximum when we collected the samples, the time duration between the collecting point and flowering time most likely represents an ecodormancy period. Thus, hormones such as IAA and jasmonate^26^, as well as epigenetic modifications such as methylation and histone modification, in this period could affect fruit yield. However, we did not observe a significant change in fruit yield between different years. Therefore, the regulation in this period should be stable and may be worthy of investigation in the future. In addition, alternative splicing is known, as a form of post-transcriptional regulation, to generate diverse isoforms^35,36^. It is possible that alternative splicing plays roles in LY and HY floral buds; however, the genome sequence, which alternative splicing analysis would require, is not currently available. Therefore, this issue will be worthy of investigation once the *C. oleifera* genome sequence is available in the future.

## Materials and Methods

### Materials

Two types of *C. chekiangoleosa* with HY and LY of fruit count were from the same location at latitude 29.978633–29.978821 and longitude 118.966596–118.967638. The trees are 20 years old with similar canopy sizes. Three individual HY trees and three LY trees were selected. The fruit number was recorded for three consecutive years for each tree. Approximately 10–15 floral buds from each tree were collected and mixed as one sample in December 2015. In total, six samples were collected for this study. The floral buds were observed to blossom in two to three months. Floral buds were frozen in liquid nitrogen immediately after collecting and kept in liquid nitrogen until the subsequent RNA extraction.

### RNA extraction and sequencing

The RNA from each sample was extracted with the methods described previously^16^. Briefly, the brown outer layers of the floral buds were removed, and the remainder was ground into fine powder. Then, the powder was used for RNA extraction, and the RNA quality and quantity were measured using agarose gels and a Nanodrop 2000c spectrophotometer (NanoDrop Technologies, Wilmington, DE, USA). The RNA integrity was measured, and then RNA-seq library was constructed as described previously^16^ and sequenced on the Illumina HiSeq 4000. The sequencing format was paired-end 125 or 150 bp. Six samples, three samples for HY and LY, were sequenced independently. The clean reads were archived in the SRA database of NCBI under the master accession number of BioProject PRJNA415233 and are publicly available.

### Transcriptome assembly, annotation and expression analysis

All clean reads were combined together to build a *de novo* transcriptome assembly for *C. chekiangoleosa*, and then all assembled unigenes, defined after clustering, were annotated against publicly available databases with the methods described previously^16^. The unigenes were archived at DDBJ/EMBL/GenBank under the accession GFZM00000000. The version described in this paper is the 2^nd^ version, GFZM02000000. The gene expression level was calculated in FPKM. The differentially expressed genes were analysed using Cufflinks (version 2.21)^37^. The DEGs were defined as having at least 2-fold change in FPKM and statistical cutoffs of *p* < 0.05 and false discovery rate *q* < 0.001 between the HY and LY groups.

For gene annotation, we used the same methods as described in our previous publication^16^. Briefly, unigenes were searched against the databases NR (NCBI non-redundant protein sequences), UniProtKB (Swiss-Prot and TrEMBL), and KOG (euKaryotic Orthologous Groups) with BLASTx^38^ with an E-value threshold of 1E-5, and against the Pfam database with HMMER3^39^. Based on all annotation IDs from UniProtKB, GO terms were retrieved from the Gene Ontology database. The pathway k number mapping was conducted using KEGG Automatic Annotation Server (KAAS, version 2.1)^21^, and pathway enrichment (hypergeometric test, *p* < 0.05 and *q* < 0.05) was conducted against *Arabidopsis* using KOBAS (version 3.0)^22^. Transcription factor and regulator analysis were conducted by using the DEG sequences as input against transcription factors in the database iTAK^23^ (http://itak.feilab.net/cgi-bin/itak/index.cgi, version 17.09) based on information from 169 plant genomes.

### Chlorophyll and leaf measurement

The chlorophyll content was measured using the chlorophyll meter SPAD-502Plus (Konica Minolta Optics, Inc.). At least 10 live leaf samples from each plant were used. For each leaf, six different spots were measured to get an average content. Leaf area and thickness were measured from 20-30 leaves with a gridded plate and calipers, respectively. The statistical T-test was used to test the significance of the differences between the HY and LY groups.

### Oil content measurement

Naturally ripened fruits were harvested from each tree in three consecutive years starting from the year 2014, and then the seeds were manually removed from the fruits after they had been naturally dried for at least two months. The hard shells were removed manually from the dried seeds. The seeds from 8–24 fruits were mechanically ground into small pieces (3 mm × 3 mm × 3 mm) and then steamed at 100 °C for 30 mins per kilogram of seeds. The oil in the seeds was squeezed with a KOMET oil pressor (Changzhou, China) with the method as described previously^40^.

## Electronic supplementary material

Supplementary information

Supplementary data
