# Supplementary material for: Comparative transcriptomic analysis identifies genes responsible for fruit count and oil yield in the oil tea plant Camellia chekiangoleosa
Source: Sci Rep. 2018 Apr 27;8:6637. doi: 10.1038/s41598-018-24073-z (PMC5923238; doi:10.1038/s41598-018-24073-z)
Supplement: Supplementary file 1 — Supplementary information [file 41598_2018_24073_MOESM1_ESM.pdf]

Supplementary information

Comparative transcriptomic analysis identifies genes responsible for fruit count and oil yield in the oil tea plant *Camellia chekiangoleosa*

Yun Xie[1], Xuewen Wang[2]

[1] Jiyang College, Zhejiang A&F University, Zhuji, Zhejiang, China, 311800, China, E-mail: 247446054@qq.com

[2] Department of Genetics, University of Georgia, Athens, 30602, USA, E-mail: xwwang@uga.edu

Table of Contents

Table S1. Trait characteristics of flowers and fruits in high and low yield C. chekiangoleosa 1

Table S2. Summary of RNA-seq reads, assembly and annotation 3

Table S3. Gene ontology term enrichment results 4

Table S1. Trait characteristics of flowers and fruits in high and low yield *C. chekiangoleosa*

	**Trait**	Flower count		Fruit count		Ratio of fruit / flower	
**Year**	replicates	LY	HY	LY	HY	LY	HY
2014	1	103	211	33	108	0.32	0.51
2014	2	58	303	21	96	0.36	0.32
2014	3	96	316	38	71	0.40	0.22
2015	1	68	203	11	117	0.16	0.58
2015	2	105	196	31	83	0.30	0.42
2015	3	71	208	25	123	0.35	0.59
2016	1	109	195	28	129	0.26	0.66
2016	2	93	301	20	91	0.22	0.30
2016	3	85	305	19	116	0.22	0.38
Mean		87.6	248.7	25.1	103.8	0.29	0.42

Standard Error		6.1	18.3	2.8	6.5	2.6E-02	5.0E-02
	Year	0.15	NOT	0.79	NOT	0.65	NOT
	Trait	9.2E-07	**	1.4E-07	**	1.0E-02	**
p value ANOVA	Interaction	0.28	NOT	0.26	NOT	0.13	NOT

"NOT" and ** represent for not or significant at probability level at 0.01 using two-way ANOVA statistical analysis. LY and HY represent the phenotypes of low yield and high yield of fruits, respectively.

Table S2. Summary of RNA-seq reads, assembly and annotation

	Category	Data					
**RNA-seq reads**	Sample ID	Low yield (A1)	Low yield (A2)	Low yield (A3)	High yield (CK1)	High yield (CK2)	High yield (CK3)
	SAR accession	SRR6199 922	SRR6199 924	SRR6199 926	SRR61999 28	SRR61999 46	SRR61999 75
	Clean reads (million)	79.630	17.024	56.986	62.941	75.300	69.384
	Q30(%)	94.8	89.9	94.3	94.6	94.9	94.9
	Total reads (million)	361					
**Assembly**							
	Total Unigenes (>200bp)				140,299		
	Total transcripts (>200bp)				238,415		
	Total length of unigene (bp)				91,583,291	485 X	
	Mean length of unigene (bp)				586		
	Count of unigenes (length >500 bp)				48,883	35.90%	
	Count of unigenes (length >1000 bp)				20,972	40.20%	
	GC%					40.0%	
**Annotation**							
	Total annotated unigenes				71,167	50.7%	
	NR (%)	non-redundant protein sequences			61,059	43.5%	
	KOG (%)	euKaryotic Ortholog Groups			41,936	29.9%	
	pfam (%)	database of protein families			41,576	29.6%	
	UniProtKB(%)	Swiss_Prot			32,356	23.1%	
	UniProtKB(%)	TrEMBL			60,225	42.9%	
	GO (%)	Gene Ontology			50,914	36.3%	

The reads were generated from Illumina Hiseq 4000 and in a format of paired-end 125 or 150 bp. The clean reads were archived at SRA database of NCBI under the master accession number of Bioproject PRJNA415233 and are

publicly available. The uni-genes were archived at at DDBJ/EMBL/GenBank under the accession GFZM00000000. The version described in this paper is the first version, GFZM02000000

Table S3. Gene ontology term enrichment results

Category	p_value	GO term	Category	Regulation	Unigenes
GO:0046686	0.0001	response to cadmium ion	bp	up	25
GO:0006096	0.0000	glycolytic process	bp	up	11
GO:0010501	0.0000	RNA secondary structure unwinding	bp	up	9
GO:0009735	0.0093	response to cytokinin	bp	up	9
GO:0051028	0.0051	mRNA transport	bp	up	7
GO:0000911	0.0153	cytokinesis by cell plate formation	bp	up	6
GO:0000398	0.0362	mRNA splicing, via spliceosome	bp	up	6
GO:0006406	0.0020	mRNA export from nucleus	bp	up	5
GO:0006974	0.0105	cellular response to DNA damage stimulus	bp	up	5
GO:0008283	0.0280	cell proliferation	bp	up	5
GO:0005829	0.0001	cytosol	CC	up	68
GO:0005886	0.0397	plasma membrane	CC	up	63
GO:0016020	0.0033	membrane	CC	up	40
GO:0005730	0.0001	nucleolus	CC	up	28
GO:0005794	0.0165	Golgi apparatus	CC	up	24
GO:0005774	0.0046	vacuolar membrane	CC	up	23
GO:0022626	0.0036	cytosolic ribosome	CC	up	13
GO:0005802	0.0090	trans-Golgi network	CC	up	11
GO:0005681	0.0104	spliceosomal complex	CC	up	9
GO:0005635	0.0046	nuclear envelope	CC	up	7
GO:0000325	0.0125	plant-type vacuole	CC	up	6
GO:0003723	0.0095	RNA binding	mf	up	19
GO:0004386	0.0001	helicase activity	mf	up	11
GO:0004004	0.0000	ATP-dependent RNA helicase activity	mf	up	10
GO:0016887	0.0159	ATPase activity	mf	up	9
GO:0000287	0.0290	magnesium ion binding	mf	up	8

GO:0004739	0.0003	pyruvate dehydrogenase (acetyl-transferring) activity	mf	up	4
GO:0003725	0.0027	double-stranded RNA binding	mf	up	4
GO:0003690	0.0129	double-stranded DNA binding	mf	up	4
GO:0050661	0.0129	NADP binding	mf	up	4
GO:0003697	0.0225	single-stranded DNA binding	mf	up	4
GO:0015074	0.0000	DNA integration	bp	down	25
GO:0015979	0.0000	photosynthesis	bp	down	18
GO:0019684	0.0000	photosynthesis, light reaction	bp	down	11
GO:0042773	0.0000	ATP synthesis coupled electron transport	bp	down	10
GO:0015986	0.0000	ATP synthesis coupled proton transport	bp	down	9
GO:0018298	0.0000	protein-chromophore linkage	bp	down	9
GO:0032197	0.0000	transposition, RNA-mediated	bp	down	7
GO:0006278	0.0000	RNA-dependent DNA replication	bp	down	7
GO:0019076	0.0000	viral release from host cell	bp	down	7
GO:0009772	0.0000	photosynthetic electron transport in photosystem II	bp	down	5
GO:0009535	0.0000	chloroplast thylakoid membrane	CC	down	57
GO:0000943	0.0000	retrotransposon nucleocapsid	CC	down	7
GO:0009522	0.0000	photosystem I	CC	down	7
GO:0009523	0.0000	photosystem II	CC	down	7
GO:0015935	0.0002	small ribosomal subunit	CC	down	7
GO:0009706	0.0009	chloroplast inner membrane	CC	down	7
GO:0009539	0.0000	photosystem II reaction center	CC	down	5

GO:0045263	0.0000	proton-transporting ATP synthase complex, coupling factor F(o)	CC	down	5
GO:0045261	0.0000	proton-transporting ATP synthase complex, catalytic core F(1)	CC	down	4
GO:0009317	0.0014	acetyl-CoA carboxylase complex	CC	down	3
GO:0003964	0.0000	RNA-directed DNA polymerase activity	mf	down	28
GO:0004190	0.0000	aspartic-type endopeptidase activity	mf	down	25
GO:0003676	0.0000	nucleic acid binding	mf	down	25
GO:0004519	0.0000	endonuclease activity	mf	down	20
GO:0048038	0.0000	quinone binding	mf	down	17
GO:0003899	0.0000	DNA-directed RNA polymerase activity	mf	down	13
GO:0008137	0.0000	NADH dehydrogenase (ubiquinone) activity	mf	down	12
GO:0003968	0.0000	RNA-directed RNA polymerase activity	mf	down	9
GO:0016168	0.0000	chlorophyll binding	mf	down	9
GO:0045156	0.0000	electron transporter, transferring electrons within the cyclic electron transport pathway of photosynthesis activity	mf	down	6

bp, CC and mf represent the biological process, cellular component and molecular function, respectively.
